# The role of extracellular vesicles and interleukin-8 in regulating and mediating neutrophil-dependent cancer drug resistance

**DOI:** 10.3389/fonc.2022.947183

**Published:** 2022-12-16

**Authors:** Mara Zippoli, Anna Ruocco, Rubina Novelli, Francesca Rocchio, Martina Sara Miscione, Marcello Allegretti, Maria Candida Cesta, Pier Giorgio Amendola

**Affiliations:** ^1^ Research and Development (R&D), Dompé farmaceutici S.p.A., Naples, Italy; ^2^ Research and Development (R&D), Dompé farmaceutici S.p.A., Milan, Italy; ^3^ Department of Biotechnological and Applied Clinical Science, University of L'Aquila, L'Aquila, Italy; ^4^ Research and Development (R&D), Dompé farmaceutici S.p.A., L’Aquila, Italy

**Keywords:** drug resistance, neutrophil, interleukin-8, extracellular vesicles, Tumor-associated neutrophils, NETosis

## Abstract

Tumor drug resistance is a multifactorial and heterogenous condition that poses a serious burden in clinical oncology. Given the increasing incidence of resistant tumors, further understanding of the mechanisms that make tumor cells able to escape anticancer drug effects is pivotal for developing new effective treatments. Neutrophils constitute a considerable proportion of tumor infiltrated immune cells, and studies have linked elevated neutrophil counts with poor prognosis. Tumor-associated neutrophils (TANs) can acquire in fact immunoregulatory capabilities, thus regulating tumor progression and resistance, or response to therapy. In this review, we will describe TANs’ actions in the tumor microenvironment, with emphasis on the analysis of the role of interleukin-8 (IL-8) and extracellular vesicles (EVs) as crucial modulators and mediators of TANs biology and function in tumors. We will then discuss the main mechanisms through which TANs can induce drug resistance, finally reporting emerging therapeutic approaches that target these mechanisms and can thus be potentially used to reduce or overcome neutrophil-mediated tumor drug resistance.

## 1 Introduction

During the past decades, huge progress has been made in the field of cancer genetics, immunology and pathology for the identification of new markers and methods for diagnosis and treatments (1, 2). Despite these achievements, resistance to classical chemotherapeutic agents or to novel drugs is one of the major causes of therapy failure and death in cancer, still representing a crucial limiting factor in the treatment of cancer patients (3).

The mechanisms through which cancer cells get resistant or acquire resistance to drug therapies are numerous, and sometimes tumors can be resistant to multiple therapies and display, simultaneously or subsequently, different mechanisms of drug resistance. In this context, it has been proposed that the mechanisms of drug resistance in tumors can be both active (cell-autonomous) or adaptive (non-cell-autonomous): the firsts depend on cancer intracellular responses, which include, for example, genetic or epigenetic alterations that promote cell survival (4–7), while the seconds result from tumor interactions with the surrounding tumor microenvironment (TME) (8, 9) that is shaped to favor tumor growth, expansion and drug resistance.

Together with myeloid-derived suppressor cells (MDSCs) and tumor-associated macrophages (TAMs), tumor-associated neutrophils (TANs) represent the most abundant population (10, 11) of immune cells infiltrated in the TME, and many studies so far have highlighted the link between elevated TAN counts and increased risk of metastasis, drug resistance and poor prognosis (12–17). In TME, neutrophils can acquire immunoregulatory capabilities, facilitating tumor progression (18) and drug resistance through a number of different mechanisms. In this context, interleukin-8 (IL-8, aka CXCL-8), a member of the CXC chemokine family that is highly produced by neoplastic cells (19), is an important chemoattractant and activator for neutrophils and is a key mediator of their biology, behavior and actions inside the tumor. On the other hand, increasing evidence is highlighting the crucial role of extracellular vesicles (EVs) in both the mediation and regulation of neutrophils’ response within the TME. EVs, produced both by tumor cells and by TANs, or by other immune or stromal cells, function in fact as intercellular mediators of the communication within the TME and beyond, and can ultimately promote neutrophil-mediated tumor drug resistance (20).

In this review, we will describe the role of IL-8 and EVs in the regulation and mediation of neutrophil biology and function in the TME, promoting pro-tumoral functions of these cells, ultimately leading to neutrophil-mediated tumor drug resistance through the production of neutrophil extracellular traps (NETs) and the secretion of neutrophil-derived EVs and other factors. Finally, we will discuss emerging therapies that, targeting IL-8, EVs and neutrophil functions, could be considered as potential therapeutic tools to reduce or overcome neutrophil-mediated tumor drug resistance.

## 2 Neutrophils in cancer

**Figure 1:**
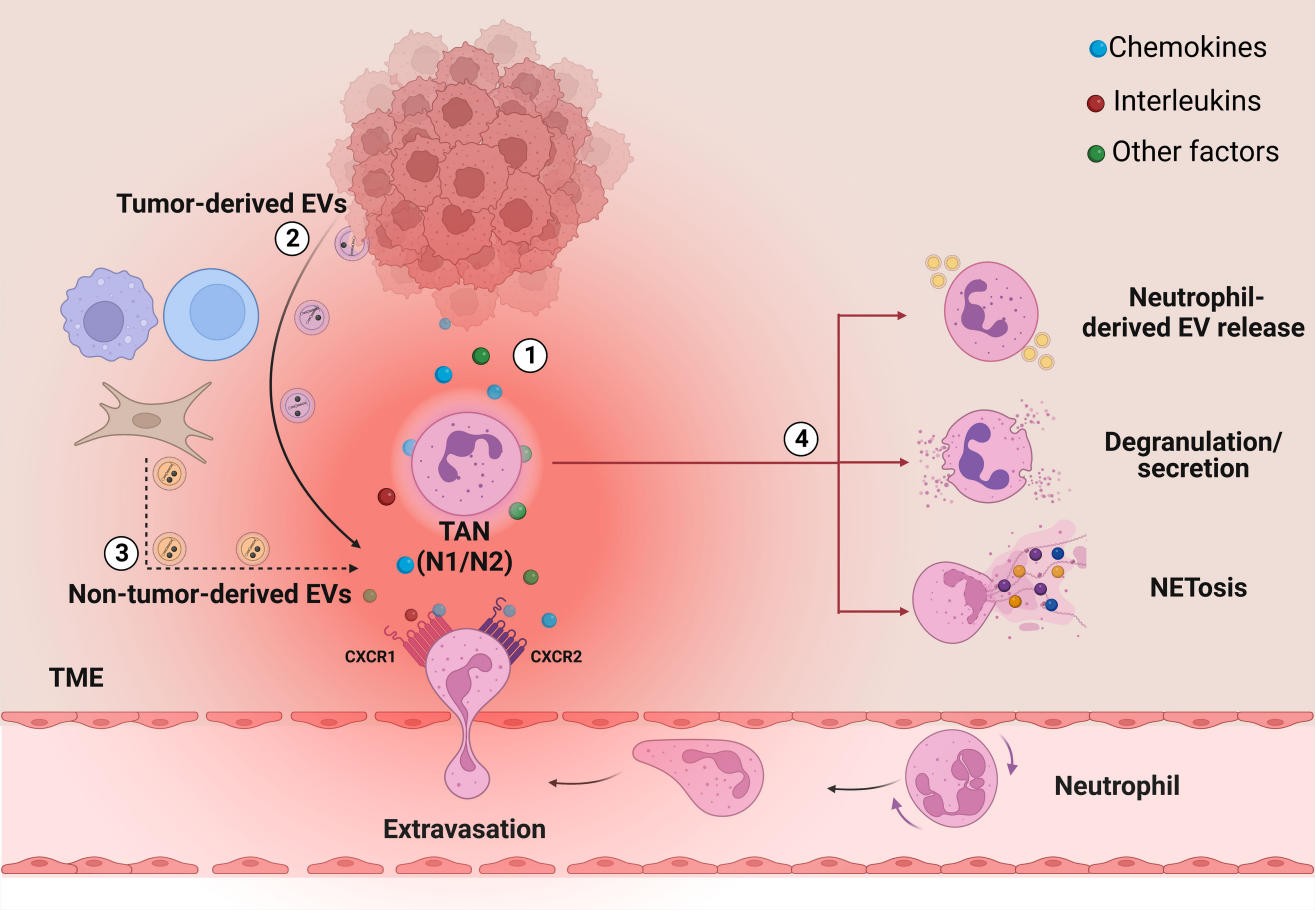
The role of secreted factors and EVs in neutrophil recruitment and activation into the TME. In TME, cancer cells can regulate neutrophil biology through the secretion of several factors, among which (1) interleukins and chemokines, such as IL-8, and (2) tumor-derived EVs. These factors regulate the recruitment of the neutrophils from the bloodstream to the tumor and the generation of TANs, which in turn promote cancer progression, metastasis and drug resistance through different cell mechanisms: release of neutrophil-derived EVs, degranulation/secretion and NETosis (4). Together with cancer cells, also other cell types in the TME can release vesicles, which are also potentially able to act on neutrophils (3, dashed arrows). Image created with biorender.com.

Neutrophils are the most abundant leukocytes in the circulation, representing around 70% of all white blood cells (21). Produced in the bone marrow (BM) through the granulopoiesis (*i.e.*, progressive maturation) of hematopoietic progenitors, neutrophils are then released into the blood stream, ready to respond to a plethora of stimuli released by inflamed tissues (22). In tumors, several chemotactic and inflammatory factors, as well as EVs, are released from tumoral and non-tumoral cells and can attract mature neutrophils, which thus migrate from blood stream and infiltrate into the TME (23–25) (**Figure 1**).

Neutrophil recruitment into the tumor site from circulation is a multi-step process that involves several factors, but seems to be mainly regulated by two G protein-coupled receptors (GPCRs): CXCR4 and CXCR2 (26). CXCR4 is a neutrophil homing marker in the bone marrow, while CXCR2 activation by its ligands (i.e., CXCL-1, CXCL-2, CXCL-3, CXCL-5, CXCL-6, CXCL-7 and CXCL-8) induces the release of neutrophils into circulation and their recruitment into the TME (27–29). Indeed, the diverse cell types in the TME (*e.g.*, tumor cells, immune cells, fibroblasts) release large quantities of CXCR2 ligands, forming a chemotactic gradient that attracts the neutrophils from the bloodstream (29). Among the chemokines that can influence neutrophil functions, IL-8 is the master regulator of neutrophil biology and one of the most characterized chemokine in cancer as it has been found overexpressed in several tumors (30–43). Once into the TME, neutrophils turn into TANs, a plastic and dynamic population that can rapidly switch between two forms: N1 TANs with anti-tumoral functions, and N2 TANs, with pro-tumoral effects (44–46). N1 TANs are mature and short-living cells, which exert their highly cytotoxic and immune-stimulating activities by producing reactive oxygen species (ROS) and other cytotoxic substances, and by recruiting and activating other immune cells (17). On the other hand, N2 TANs are immature and long-living cells, which can produce and release cytokines, chemokines and other factors to favor pro-angiogenic, pro-metastatic and immune-suppressive activities (47). TANs polarization towards one of the two sub-populations is crucially regulated by multiple TME factors including, among others, cytokines and chemokines, such as IL-8, and also EVs, released by tumor, stromal and immune cells (17, 48, 49). In addition, TANs can also regulate cancer progression through NETosis, a process by which neutrophils extrude a sort of web-like structures called NETs (50–52). NETs are formed by DNA fibers decorated with cytotoxic enzymes, such as neutrophil elastase (NE), myeloperoxidase (MPO) and matrix metalloproteinases-9 (MMP-9) and are released by activated neutrophils into the extracellular space as mechanism of defense against pathogen micro-organisms (53). In tumors, NETs have been identified as factors that can significantly contribute to carcinogenesis and metastasis (11, 54) in several ways, as by inducing the degradation of the extracellular matrix which promotes the extravasation of cancer cells (50), trapping circulating tumor cells (CTCs) (55, 56) or deactivating thrombospondin-1 (TSP-1), a potent inhibitor of angiogenesis and tumor progression (57, 58).

Regulated by different factors and acting through several mechanisms, neutrophils thus play a key role in hijacking the immune system response against the tumor, ultimately promoting cancer progression and tumor drug resistance (59, 60).

## 3 IL-8 and EVs crucially regulate and mediate the biology and functions of TANs in the TME

### 3.1 IL-8 and TANs

Tumor cells produce several factors, such as cytokines, chemokines, lipids, and growth factors that, not only increase their growth and survival in an autocrine manner (61), but also increase the number of circulating neutrophils by stimulating granulopoiesis in the bone marrow and promote their recruitment to TME in a paracrine manner (62–64) (**Figure 1**). In particular, among other factors, IL-8 has demonstrated to be crucial in tumor progression (19, 65–67), since it was found to be overexpressed in several tumors, where induces angiogenesis and is involved in the maintenance of cancer stem cells (CSCs) (68, 69). Also, a direct correlation between IL-8 and poor prognosis has been reported (70–73). IL-8 exists as a monomer or dimer and exerts its activity by binding its two receptors: CXCR1 and CXCR2 (74). It is a well-known chemoattractant able to recruit leukocytes and in particular neutrophils, which express a substantial number of IL-8 receptors on their surface (75, 76). During carcinogenesis, the IL-8 released by neoplastic cells promotes the activation of both the phosphatidylinositol-3-kinase (PI3K) and the mitogen-activated protein kinase (MAPK) signal pathways *via* CXCR2, thus leading to cell migration and survival (77–79). In addition, IL-8 mediates the formation of NETs, through the binding to CXCR1 and CXCR2 (18, 80) (**Figure 1**). These mechanisms help to dampen the anti-tumor immune responses and cause disfunctions of cytotoxic immune cells, thus crucially contributing to tumor growth and progression (81).

### 3.2 Extracellular vesicles and TANs

In addition to IL-8, EVs are other factors that are crucially involved in the regulation and mediation of TANs’ pro-tumoral functions in the TME (82). EVs are heterogenous lipid bilayer structures secreted by cells that can carry a plethora of cargoes, including lipids, proteins and nucleic acids (83–86). In the past they were divided in three subtypes (microvesicles (MVs), exosomes and apoptotic bodies) depending on their biogenesis, release pathways, size, content and functions (84, 87, 88). However, since it is not easy to clearly determine EVs biogenesis pathway, the last MISEV guidelines (MISEV 2018) suggest to classify EV subtype referring to 1) physical characteristics of EVs, such as size or density; 2) biochemical composition; or 3) descriptions of conditions or cell of origin (89). One of the main functions of EVs is to facilitate the exchanges of cellular components, acting as an intercellular communication system in both physiological and pathological conditions (88, 90, 91). The EV-mediated intercellular communication is achieved in two manners: by delivering cargoes that are within the vesicles in the target cells (92, 93), or by using EVs surface markers without requiring vesicle internalization (94). In tumors, EVs are important components of the TME and promote the crosstalk between cancer and cancer-associated cells (e.g. fibroblasts, endothelial and immune cells), creating a favorable niche that supports and nourishes the tumor, promoting its growth and progression, and also regulating tumor drug resistance.

Tumor-derived EVs are nanoscale membrane vesicles (95) that contain tumor-specific functional biomolecules both in their lumen, such as cytokines, growth factors, proteases and enzymes, as well as on their surface, including receptors/ligands, adherent molecules, or tetraspanins (96, 97). Tumor-derived EVs work in both autocrine and paracrine way to favor local invasion of tumor cells and spreading of metastasis and to induce the reprogramming of recipient cells (82, 98–101). They can also promote immune-modulation by attenuating the cytotoxic activity of T and NK cells, prompting the recruitment of regulatory B cells and Tregs and inducing the differentiation of M2 macrophages and N2 immune-suppressive sub-population of tumor-associated macrophages (TAMs) and TANs, respectively (102, 103), thereby creating a pro-tumorigenesis environment for tumor progression (104–106).

Among innate immune cells, neutrophils may be especially prone to stimulation from tumor-derived EVs (107); for example, they can promote TAN polarization into the anti-inflammatory N2 tumorigenic subtype (**Figure 1**). Although the underlying mechanisms remain poorly understood, Zhang and colleagues have recently started analyzing tumor-derived EVs induced N2 neutrophil polarization in gastric cancer, demonstrating that gastric cancer-derived EVs can induce the expression of programmed death-ligand 1 (PD-L1) on neutrophils, which in turn polarizes their differentiation through the N2 phenotype and suppresses T cell-mediated immunity (108, 109). On the other hand, tumor-derived EVs from murine colorectal CSCs have been shown to prolong bone marrow-derived neutrophil life-span through the activation of the NF-κB signaling, which in turn induces the expression of interleukin-1β (IL-1β) in neutrophils, thus promoting their pro-tumoral phenotype (110). Besides inducing N2 polarization, tumor-derived EVs can also modulate other properties of neutrophils’ biology. Tumor-derived EVs from a metastatic human melanoma cell line (MV3), for example, have been shown to induce neutrophil chemotaxis through the CXCR2/PI3K-Akt axis and to promote the formation of NETs (103) (**Figure 1**), which play a crucial role in inducing cancer-associated thrombosis (111–113) and tumor drug resistance (114). Similar results have been obtained in a mouse model of breast cancer, where 4T1-derived exosomes induced NETs formation in neutrophils derived from G-CSF-treated mice and accelerated venous thrombus formation in tumor-free neutrophilic mice (115). Also the EVs released from a human cell line of breast carcinoma (MDA-EVs) induced neutrophil activation (*i.e.*, increased chemotaxis and secretion of IL-8 and MMP-9), N2-like phenotype and increase of ROS production, which were followed by augmented NETosis (116). Finally, a recent report also showed that exosomes can transfer mutant KRAS from DKO-1 colorectal cancer cells to neutrophils, resulting in increased IL-8 production, neutrophil recruitment and NETs formation, ultimately promoting tumor growth and metastasis. Interestingly, these effects were abolished by an anti-IL-8 treatment (117).

Although tumor-derived EVs represent the majority of vesicles secreted in the TME, studies have shown that EVs can be released also by other cells within the TME, such as cancer-associated stromal cells (CASCs), including fibroblasts, immune cells, endothelial cells and neurons (118) (**Figure 1**). These EVs can influence many aspects of tumor biology, but their direct role in the regulation of neutrophil biology has not been fully addressed yet. For example, cancer associated fibroblasts (CAFs) can secrete EVs which act on cancer cells to enhance their metastatic potential by delivering bioactive molecules, such as extracellular matrix proteins and remodeling enzymes (118). Ji et al. demonstrated that primary colorectal cancer cells can secrete integrin beta-like 1 (ITGBL1)-bearing EVs which enter the circulation, reach distant organs, and activate fibroblasts *via* the TNFAIP3-mediated NF-κB signaling (119). In addition to fibroblasts, also immune cells can release EVs within the TME ultimately exerting anti-cancer effects as for natural killer (NK) cells, or pro-cancer effects in the case of regulatory T cells (Tregs) (120). NK cell-derived EVs are released by resting and activated NK cells and both can exert cytotoxic activity on activated but not resting immune cells (121), but also exhibit immune-modulatory activity by stimulating other immune cells *via* paracrine action or through the circulatory system (122).

Further studies are needed to better understand if this subset of non-tumor-derived EVs may have a direct role in the regulation of neutrophil biology in the context of tumor progression.

## 4 TAN-mediated tumor drug resistance

The involvement of neutrophils in tumor drug resistance is determined by the interplay of several factors. Among others, IL-8 and EVs are key modulators of neutrophil biology and functions within the TME. They act on neutrophils to promote tumor drug resistance which is exerted through different mechanisms, such as release of neutrophil-derived EVs, secretion of specific molecules/factors and NETosis (**Figure 1**). These mechanisms can act in a concerted way to promote tumor drug resistance by reducing the availability or stability of administered therapeutics, inducing ROS production or alterations of DNA damage repair pathways, and modulating antitumor immunity (123).

### 4.1 Tumor drug resistance promoted by neutrophil-derived EVs

Like tumor cells, neutrophils can also produce and release EVs in response to intracellular metabolic changes and/or extracellular environmental stress. As reviewed by Rubenich and colleagues, the genetic and molecular composition of neutrophil-derived EVs reflects that of the mother cell and varies depending on the existing physiological or pathological conditions (20, 124). Depending on the context, neutrophils polarize into inflammatory N1 or regenerative N2 subtypes, which are thought to be able to release two different kinds of EVs: the N1-derived and the N2-derived EVs, respectively (124).

**Figure 2:**
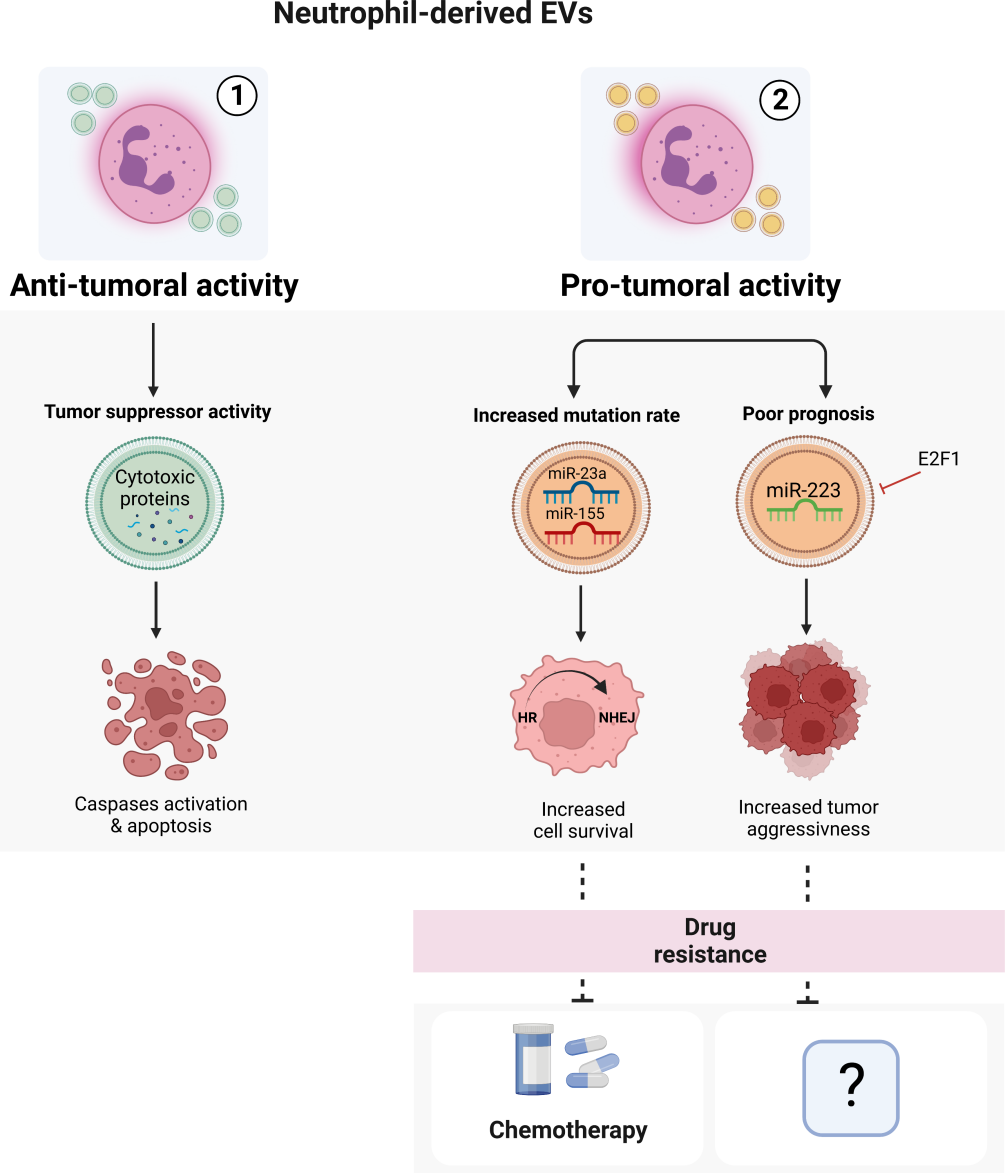
Neutrophil-derived EVs. Neutrophil-derived EVs can exert both anti-tumoral (1) or pro-tumoral (2) activities in a context-dependent manner. The EVs isolated from healthy neutrophils can induce apoptosis of cancer cells through the activation of caspases pathway. On the other hand, tumoral neutrophil-derived EVs seem to promote cancer spreading, progression and drug resistance. Image created with biorender.com.

During cancer progression, the role of neutrophil-derived EVs seems to be important for the prediction of disease outcome, although the underlying mechanisms are still unclear (125). Even if few, the available evidences on neutrophil-derived EVs isolated from tumoral contexts seem to mainly suggest a role for these vesicles in mediating cancer progression and drug resistance. On the other hand, EVs produced by neutrophils from healthy donors may possess a tumor suppressive activity both *in vitro* and *in vivo* (126). As recently demonstrated in fact, EVs from healthy neutrophils contain cytotoxin proteins that are able to activate the caspases signaling pathway and then promote tumor cell apoptosis (126) (**Figure 2**).

The role of neutrophil-derived EVs in drug resistance has been demonstrated by a recent work from Butin-Israeli and colleagues (127). Using samples from inflammatory bowel disease (IBD) patients, who are more prone to develop colitis-associated colorectal cancer and have an important neutrophil infiltrate in the intestinal mucosa, they demonstrated that neutrophil-derived EVs containing miR-23a and miR-155 inhibited Homologous Recombination (HR) repair by targeting the main HR regulators RAD51 while promoting non-homologous DNA end joining (NHEJ), ultimately leading to the formation of highly mutagenic DNA Double-Strand Breaks (DSBs) (127). This switch from HR to NHEJ may result in the acquisition of drug resistance in tumors (128–131) as observed in colorectal cancer, in which neutrophil-mediated NHEJ induced resistance to a lethal dose of topo-isomerase I inhibitor Camptothecin (CMPT) as tumor cells effectively resolved CMPT-induced DSBs and entered normally into cell cycle (132) (**Figure 2**). Other evidence for the role of neutrophil-derived EVs in cancer, suggest that they can act either as an onco-suppressor (133–136) or as an onco-promoter (137–139) in a context-dependent manner. For example, neutrophil-derived EVs containing miR-223, a miRNA essential for the development of cells of the myeloid lineage and the mobilization of neutrophils from the bone marrow (140–142), have been described to be able to both sustain and inhibit tumor growth (135, 137–139, 143). In both acute myeloid leukemia and breast cancer for instance, E2F1-dependent downregulation of EVs-transported miR-223 is associated with tumor aggressiveness and poor prognosis (135, 143). Of note, a clear role of neutrophil-derived EVs carrying miR-223 in drug resistance still remains unknown (**Figure 2**).

Interestingly, in addition to regulate tumor progression and drug resistance, neutrophil-derived EV have recently also been engineered to efficiently deliver anti-cancer drugs at the tumor site (126), thus not only demonstrating the intricate complexity of the processes regulating neutrophil-derived EVs content and secretion but also showing the therapeutic potential of these vesicles.

### 4.2 Tumor drug resistance promoted by TAN-released factors and NETosis

**Figure 3:**
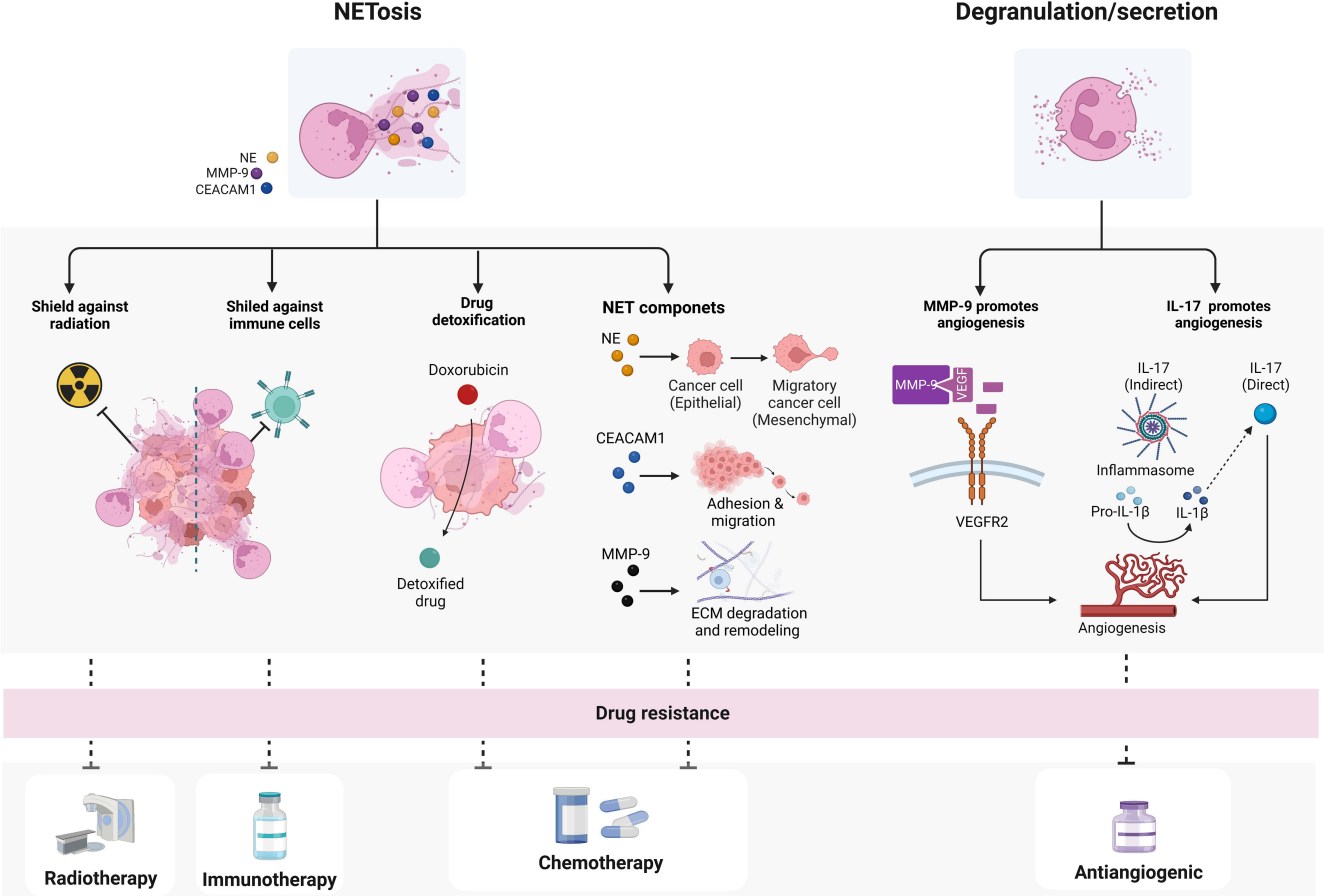
Mechanisms by which TANs may confer drugs resistance. TANs can promote drug resistance through two main mechanisms: NETosis and degranulation/secretion. NETs or NETs components can mediate resistance to immune- radio- and chemotherapy, while neutrophil-secreted factors have been shown to mainly influence angiogenesis and interfere with angiogenic therapies. biorender.com.

Attracted to the tumor site and regulated by the action of IL-8, EVs and other chemotactic factors, TANs can interfere with different antitumoral treatments not only by releasing EVs but also by secreting specific factors as well as by undergoing NETosis. During degranulation and NETosis, TANs can for example increase the secretion of matrix metalloproteinases (MMPs), such as MMP-2 and MMP-9, thus counteracting the effects of anti-angiogenic therapies. MMP-9, the production of which is also directly induced by IL-8 through CXCR2 receptor (144), can in fact cleave matrix-bound isoforms of VEGF-A into soluble fragments that are able to elicit VEGFR2 receptor activation and induce angiogenesis with a higher potential than uncleaved protein (145, 146) (**Figure 3**). In addition, TANs can directly secrete the pro-angiogenic cytokine IL-17 (147) or induce the activation of cathepsin B/NLRP3 inflammasome followed by IL-1β overproduction, with consequent increase of IL-17 secretion (148, 149) (**Figure 3**).

Besides secreting factors in the TME, TANs can mediate drug resistance also through the formation of NETs or through the activities of several NET-associated components (**Figure 3**). In agreement with this, increased levels of cell free cell free DNA (cfDNA), which is at least in part derived from NETs, predict limited response to chemo- and immune-therapy in several tumors (150–152). NET components, including NE, MMP-9, Cathepsin G (CG), the carcinoembryonic antigen cell adhesion molecule 1 (CEACAM1), and other factors, have been shown to promote resistance to chemotherapy through different mechanisms (55, 153–156). Preclinical studies suggest that NE can promote malignancy and resistance to chemo- and immune-therapy by inducing cell epithelial-mesenchymal transition (EMT) (153, 154, 157). Evidence emerged to support the infiltration of neutrophils into TME as a driver of EMT through NE activity (158–160). On the other hand, MMP-9 and CG, associated with NETs, mediate the degradation and remodeling of the extracellular matrix and, as discussed above, promote angiogenesis, so that their presence has been associated with tumor progression and poor response to chemotherapy (155, 161). Finally, CEACAM1 protein, that decorates NETs and facilitates NET-dependent pro-metastatic interactions by improving neoplastic cells adhesion and migration, is potentially involved also in mediating cancer response to therapy (156) (**Figure 3**).

Increased NETosis promotes tumor resistance also to radiation-therapy (RT) (162). In a syngeneic bladder cancer model, RT increased NET deposition and, notably, when NETosis was inhibited by DNase I or neutrophil elastase inhibitor, the overall radiation response improved. Consistently with these data, NETs have been also observed in bladder tumors of patients who did not respond to RT and had persistent post-RT relapse (163, 164) (**Figure 3**). In addition, tumor-associated NETs can also support metastatic cells to evade immune response by creating a physical shield from cytotoxic immune cells, such as cytotoxic CD8+ T and natural killer cells (NKs), thus preventing interactions between tumor and effector immune cells (165, 166) (**Figure 3**). In line with this, NETs formation has also been shown to mediate the resistance to checkpoint blockade, thus reducing responses to immunotherapy (18, 167–169). NETs can also have a role in detoxifying tetracycline drugs, such as doxorubicin (**Figure 3**), and degradation of NETs through DNase treatment restored chemosensitivity in animal models, demonstrating a functional role for NETs in chemo-resistance (166). Although this finding has yet to be corroborated in other tumors, this emerging evidence is notable since it raises NETs as therapeutic targets for the improvement of chemotherapy response.

Pharmacological NETosis inhibition has been shown to synergize with immunotherapies, such as anti-PD-1 and anti-CTLA-4 mAbs (18, 170), possibly by favoring cytotoxic effector T cell response against cancer cells following checkpoint inhibition. As further confirmation of the role of NETosis in immunotherapy resistance, it has been demonstrated that hPMNs recruited by IL-17 in pancreatic ductal adenocarcinoma undergo NETosis, and when NETosis is abrogated, the tumor acquires an immunotherapy-sensitive phenotype (171).

In conclusion, TANs and related regulatory factors and mediators (*i.e.*, IL-8, EVs, and other secreted factors) represent potential targets for novel therapeutic approaches aiming to target cancer cells and reduce drug resistance.

## 5 Therapeutic strategies to inhibit neutrophils in cancer progression and cancer drug resistance

### 5.1 Investigational drugs

#### 5.1.1 Targeting CXCR1/2 and neutrophils

With the aim to overcome the deleterious effects of neutrophils in cancer, the IL-8 and CXCR1/CXCR2 inhibition could reduce neutrophils migration to the tumor, thus avoiding NETs formation and eventually preventing drug resistance. In this section, we briefly report an overview of investigational drugs targeting IL-8 and its receptors CXCR1/CXCR2, and discuss their therapeutic potential in the field of cancer resistance (**Table 1**).

**Table 1:** Summary of the main CXCL8-CXCR1/2 inhibitors for cancer therapy.

Drug	Therapeutic combination	Indication	Trial phase/Study type	Recruitment status	NCT number
	Nivolumab (anti PD-1)	Advanced solid tumor	Phase 1	Completed	NCT02536469
	Cabiralizumab (anti CSF1R)	Head and neck squamous cell carcinoma	Phase 2	Recruiting	NCT04848116
Humax IL8	Nivolumab (anti PD-1)	Prostate cancer	Phase 1	Recruiting	NCT03689699
	Nivolumab (anti PD-1)	Adenocarcinoma of the prostate	Phase 2	Recruiting	NCT03689699
	Nivolumab (anti PD-1)	Pancreatic cancer	Phase 2	Recruiting	NCT02451982
Navarixin	Pembrolizumab (anti PD-1)	Metastatic solid tumor	Phase 2	Completed	NCT03473925
	Durvalumab (anti PD-L1)	Metastatic pancreatic ductal carcinoma	Phase 1/2	Completed	NCT02583477
AZD5069	Durvalumab (anti PD-L1)	Advanced solid tumor and squamous cell carcinoma of head and neck	Phase 1/2	Active, not recruiting	NCT02499328
	Monotherapy	Myelodysplastic syndrome	Phase1	Recruiting	NCT04245397
SX-682	Pembrolizumab (anti PD-L1)	Metastatic melanoma	Phase 1	Recruiting	NCT03161431
	Nivolumab (anti PD-1)	Metastatic colorectal cancer	Phase 1/2	Recruiting	NCT04599140
	Nivolumab (anti PD-1)	Metastatic pancreatic ductal adenocarcinoma	Phase 1	Recruiting	NCT04477343
Reparixin	Monotherapy	Fatigue	Phase 2	Not yet recruiting	NCT05212701
		Locally advance or metastatic breast cancer			
	Paclitaxel (antineoplastic agent)	Metastatic breast cancer	Phase 1	Completed	NCT02001974
	Paclitaxel (antineoplastic agent)	Metastatic breast cancer	Phase 2	Completed	NCT02370238
	Monotherapy	Breast cancer	Phase 2	Terminated	NCT01861054
RP-72	Monotherapy or combination with gemcitabine	Pancreatic cancer	Phase 1	Recruiting	NCT04338763

HuMax-IL8, also known as BMS-986253, is a fully human monoclonal antibody inhibitor of the IL-8 pathway. Humax-IL-8 was shown to block tumor progression (172), immune escape, EMT and MDSCs recruitment (173) in humans, thus pushing further new investigations in cancer resistance (172). HuMax-IL8 was developed for the treatment of patients with advanced solid tumors in combination with nivolumab, an anti-PD-1 monoclonal antibody immune check point inhibitor (NCT02536469), and it is currently under clinical evaluation for the treatment in several other tumors, including advanced solid tumors (NCT03400332), non-small cell lung cancer (NSCLC) (NCT04123379), advanced melanoma and metastatic renal cell carcinoma (NCT04050462), pancreatic cancer (NCT02451982), and head and neck squamous cell carcinoma (NCT04848116). In addition, HuMax IL8 is currently in phase 1b/2 trial in combination with nivolumab for treatment of men with hormone-sensitive prostate cancer (NCT03689699).

Navarixin is a CXCR1/CXCR2 receptor antagonist that impairs neutrophils recruitment (174), and that was shown to repress tumor cells metastasis and angiogenesis in preclinical models (175, 176). The molecule was shown to suppress CXCR2 signaling by decreasing MAPK/AKT pathway phosphorylation, resulting in sensitization of colorectal cancer cells to oxaliplatin treatment (177). Navarixin was assessed for its efficacy and safety in combination with pembrolizumab, an anti-PD-1 monoclonal antibody, in a phase 2 clinical trial of three types of solid tumors: programmed death-ligand 1 (PD-L1) positive refractory non-small cell lung cancer (NSCLC), castration resistant prostate cancer (CRPC) or microsatellite stable (MSS) colorectal cancer (CRC) (NCT03473925).

AZD5069 is a reversible CXCR2 antagonist that was shown to inhibit IL-8 or GRO-α-induced cytosolic calcium increase, CD11b surface expression, adhesion and chemotaxis in neutrophils (178, 179). The molecule was developed as part of combination therapies with durvalumab, an anti PD-L1 monoclonal antibody, in cancer indications including metastatic squamous cell carcinoma of the head and neck (SCCHN) (NCT02499328), and pancreatic ductal adenocarcinoma (NCT02583477).

SX-682 is a CXCR1/CXCR2 antagonist with potential anticancer activities. It exhibited significant activity in solid tumor models, where it reversed chemoresistance and extended overall survival. In syngeneic and genetically engineered mouse (GEM) melanoma models, it potently synergized with anti-PD1 therapy inducing complete remissions (180). In addition, it enhanced both PD-1 immune check point blockade, reduced MDSCs in the TME, and increased natural killer (NK) and T cells infiltration into the tumor site in animal models of head and neck tumor (181). The molecule is currently under active development as monotherapy or in combination with anti PD-1 molecules for the treatment of myelodysplastic syndrome (MDS) (NCT04245397), melanoma (NCT03161431), metastatic colon adenocarcinoma or colorectal carcinoma (NCT04599140) and metastatic pancreatic adenocarcinoma (NCT04477343).

Reparixin is an antagonist of IL-8 that binds CXCR1 and CXCR2 receptors to prevent neutrophil chemotaxis, thus avoiding graft tissue damage in organ transplantation and cancer, including breast cancer (182, 183). The combination of reparixin with antineoplastic agent docetaxel reduced the tumor size in a model of human breast cancer cell lines and breast cancer patient-derived xenografts (184) demonstrating that reparixin is able to reduce *in vivo* the tumor-initiating ability of breast cancer cells by affecting the CSC population; in fact, in tumor-bearing mice treated with reparixin alone or in combination with chemotherapy, the CSCs proportion was far lower than in tumor from mice receiving chemotherapy alone. Additional preclinical evidence highlighted the antitumor and antistemness activity of reparixin in epithelial thyroid cancer (185) and pancreatic cancer (186). Several clinical trials were conducted to assess the efficacy of reparixin in combination with taxanes or in monotherapy in metastatic breast cancer (NCT02001974, NCT02371238, NCT0161054). A new phase 2 clinical trial (NCT05212701) has started to evaluate the efficacy of reparixin in the treatment of oncological fatigue in locally or advanced metastatic breast cancer, a highly disabling condition, very common in cancer patients.

Danirixin is a CXCR2 antagonist originally developed for the potential oral treatment of chronic pulmonary disease (COPD). The molecule is able to strongly reduce the CD11b upregulation mediated by IL-8 or GRO-α agonists in healthy donor neutrophils, thus making the molecule a potential therapeutic agent for diseases characterized by neutrophil hyperactivation (187). In addition, Danirixin was found to block migration, invasion and EMT events mediated by TAMs and IL-8 in a preclinical *in vitro* model of breast cancer (188).

RP-72 is a 72 amino-acid recombinant protein that blocks the activation of IL-8-mediated signaling transduction pathways by decreasing proliferation of susceptible pancreatic cancer cells. The protein is under a Phase 1 clinical trial development for the potential intravenous treatment of metastatic pancreatic cancer in monotherapy or in combination with antiangiogenic gemcitabine (NCT04338763).

#### 5.1.2 Targeting EVs

Targeting EVs in cancer progression could also represent a good strategy to counteract tumor drug resistance (**Table 2**). In this context, promising results were obtained in an *in vitro* model of ovarian cancer, in which the treatment with heparin, amiloride and dynasore inhibited EV release after treatment with cisplatin (189) known as mechanism responsible for cancer resistance to the therapy. Similar results were obtained in another model of ovarian cancer, in which the phospholipase inhibitor GW4869 was shown to inhibit the exosomal DNA methyltransferase 1 (DNMT1)-mediated cisplatin resistance in cells, and to increase apoptosis (190). These findings suggest that the combination of cisplatin with EV inhibitors can potentially overcome the drug resistance. In a melanoma model, the same GW4869 inhibited exosome secretion that caused the induction of tumor cell proliferation and apoptosis (191). A similar effect was observed in a model of prostate cancer where treatment with GW4869 effectively reduced cancer cell viability associated to exosome secretion (192). In aggressive B-cell lymphomas, suppression of exosomal drug resistance with indometacin increased efficacy of doxorubicin therapy (193). Finally, in a tumor mice model the treatment with dimethyl amiloride (DMA), known to reduce exosome release into the bloodstream, given in combination with the chemotherapeutic drug cyclophosphamide, halted the tumor growth by 50% or more, if compared to the untreated controls (192).

**Table 2:** Summary of the main anti-EV agents in cancer and cancer drug resistance in preclinical models.

Drug	Antitumor therapy	Mechanism of targeted or cancer therapy resistance	*In vitro* model	Reference
Heparin	cisplatin	EV uptake inhibitor	Ovarian cancer	Samuel P et al., 2018 (189)
Amiloride	cisplatin	EV uptake inhibitor	Ovarian cancer	Samuel P et al., 2018 (189)
Dynasore	cisplatin	EV uptake inhibitor	Ovarian cancer	Samuel P et al., 2018 (189)
GW4869	cisplatin	EV inhibitor	Ovarian cancer	Cao Y et al., 2017 (190)
		EV inhibitor	Melanoma	Matsumoto A et al., 2017 (191)
		EV inhibitor	Prostate cancer	Panigrahi GK et al., 2018 (192)
Indomethacin	doxorubicin/pixantrone	EV inhibitor	Lymphoma	Koch R et al., 2016 (193)

Thus, new interest is arising for the development of EV/exosome pathway inhibitors. The combined use of IL-8 biological activity inhibitors that modulate the hyperactivation of neutrophils could represent a new strategy to mitigate cancer drug resistance induced by EVs release. A first example of such approach is represented by the combined blockade of IL-8 and IL-6 in osteosarcoma. Starting from data showing that osteosarcoma tumor-secreted EVs can induce a pro-metastatic phenotype by strongly inducing IL-6 production in mesenchymal stem cells (MSCs), it has been demonstrated that EVs from aggressive cancer cell lines can induce MSCs to express inflammatory cytokines and chemokines, among which IL-8 was the most upregulated one, and that this was due to tumor EV-associated non-coding RNAs. The blockade of IL-8 signaling with ladarixin (an allosteric inhibitor of CXCR1 and CXCR2) and, even more strikingly, its combination with tocilizumab (an anti-IL-6 receptor antibody) reduced lung metastasis formation in a xenograft mouse model of osteosarcoma and, notably, prevented the occurrence of MSC-induced tumor resistance to antimetastatic drugs (abstract submitted to the ASCO 2022 meeting).

## 6 Conclusions

TANs play a key role in tumor drug resistance, and their activities in this context are regulated and mediated by different factors. Among these, EVs and IL-8, produced either by tumoral cells or by neutrophils themselves, crucially function to both control and mediate the pro-tumoral functions of neutrophils in the TME. The role of both EVs and IL-8 is crucial for neutrophil-mediated tumor drug resistance, which is mainly due to the induction of NETs formation and the secretion of pro-tumoral factors, including neutrophil-derived EVs. Growing evidence has highlighted the close association between high levels of IL-8, EVs production, NETosis, and limited therapeutic response in a variety of malignancies, thus paving the way to investigations on the therapeutic potential of combination treatments either of IL-8 activity blockers, or anti-EVs drugs, or NETosis inhibitors with standard antitumoral therapies, to reduce or counteract tumor drug resistance (162, 193).

In conclusion, IL-8 and EVs represent key potential targets for the development of novel therapeutic options aimed to target neutrophil-mediated tumor drug resistance.

## Author contributions

MZ, AR, FR, and MSM performed data collection (literature reviewing) and prepared the original draft of the manuscript. RN revised and wrote the final version of the manuscript. MA and MCC revised the manuscript for critically important intellectual content. PGA conceptualized the study and revised and wrote the final version of the manuscript. All authors contributed to the article and approved the submitted version.
